# Positive death attitudes and psychological well-being: examining the influence of the four immeasurables

**DOI:** 10.3389/fpubh.2025.1622813

**Published:** 2025-06-27

**Authors:** Alla Glushich, Nahathai Wongpakaran, Tinakon Wongpakaran, Justin DeMaranville, Danny Wedding

**Affiliations:** ^1^Mental Health Program, Multidisciplinary and Interdisciplinary School, Chiang Mai University, Chiang Mai, Thailand; ^2^Department of Psychiatry, Faculty of Medicine, Chiang Mai University, Chiang Mai, Thailand; ^3^Department of Clinical and Humanistic Psychology, Saybrook University, Pasadena, CA, United States; ^4^Department of Psychology, University of Missouri-Saint Louis, St. Louis, MO, United States

**Keywords:** well-being, self-esteem, death attitudes, prosociality, older adults

## Abstract

**Background:**

The global ageing population trend has heightened the need to understand various factors affecting geriatric well-being, including beliefs and attitudes about death. Positive perspectives about death, such as those gained from practicing maranasati (death contemplation in Buddhism), may enhance psychological resilience and prosocial behavior, key factors promoted in public mental health. Death reflection can encourage the development of the Four Immeasurable Minds (equanimity, loving-kindness, compassion, and sympathetic joy), Buddhist virtues that promote public harmony and enhance well-being.

**Objective:**

The current study investigated whether positive beliefs and attitudes about death influenced well-being directly or indirectly through the Four Immeasurable Minds among older Thai Buddhists, contributing to culturally informed mental health strategies.

**Materials and methods:**

Data were collected from 332 older adults in Northern Thailand. The study collected information on positive beliefs and attitudes about death using the Morana Questionnaire, well-being using the WHO-5 index (Thai), and Four Immeasurable Minds using the Four Immeasurable Inventory. The data were analyzed using mediation analysis, accounting for potential confounding variables.

**Results:**

Positive beliefs about death had a significant and positive effect on well-being, and prosocial values mediated this relationship.

**Conclusion:**

The findings suggest that positive beliefs about death can promote prosocial values and contribute to a healthy sense of well-being among Buddhist Thai older adult. Integrating culturally adapted, mindfulness-based approaches into public mental health initiatives could support healthy aging, particularly in Buddhist communities. Further research should explore scalable interventions to promote psychological resilience in diverse older populations.

## Introduction

The global aging trend has heightened awareness of the well-being of older adults, prompting investigations into factors that contribute to their quality of life (1). Older individuals often face challenges such as loneliness and physical limitations, which can hinder personal fulfillment and community participation (2). As a result, promoting their well-being has become a crucial public health concern. The World Health Organization (WHO) defines well-being as encompassing quality of life and the ability of individuals and societies to engage with the world with meaning and purpose. It is influenced by both objective factors, such as employment and education (3), and subjective factors, including personality traits and attitudes (4).

Among these influences, death attitudes play a critical role, shaping an individual’s perception of life, self, and social relationships (5). However, much of the existing research on death attitudes focuses on death anxiety and its negative consequences. In contrast, positive death attitudes and their potential benefits for mental health are often overlooked (6). Given that beliefs about death —and responses to its reminders— are primarily shaped by the sociocultural and religious contexts, this trend may stem from the negative framing of death in the modern Western discourse (7). However, such perspectives are not universal.

Previous research identifies several death attitudes, including fear of death, death avoidance, escape, death acceptance, neutral acceptance, and approach acceptance (8). The latter two are often associated with better mental health outcomes and are generally considered more adaptive from a psychological perspective (9). Approach acceptance refers to a belief in a better afterlife. In contrast, neutral acceptance encompasses broader perspectives, such as viewing death as a natural part of life, embracing one’s cultural identity, and living a meaningful existence. Adopting death-accepting attitudes can lead to personal transformation, fostering prosocial behavior, a greater appreciation of life and relationships, and enhanced self-worth (5).

In Theravada Buddhism, the predominant religion of Thailand, with over 90% of the population identifying as Buddhist (10, 11)— Death is regarded as a natural and inevitable phenomenon. Within this tradition, life and death are viewed as part of an interdependent cycle, where one’s actions and attitudes toward death influence one’s future rebirth. This perspective aligns closely with neutral and approach acceptance attitudes and is associated with greater psychological well-being (12). A study of older Thai adults similarly found evidence of death-accepting attitudes (13).

These attitudes are cultivated through contemplation of death, or maranasati, a Buddhist meditation practice that encourages reflection on the nature of death and impermanence. As one of Buddhism’s 10 recollections (‘marana’ meaning death + ‘sati’ meaning mindfulness/awareness), maranasati is included among the 40 subjects of meditation. It is a deliberate and structured practice, undertaken in an appropriate mental state and under the right conditions, allowing individuals to reflect on their mortality. Practitioners are encouraged to examine obstacles to higher rebirth, motivating engagement in Buddhist practices such as meditation and personal growth through the Four Immeasurables (Brahmavihāra)—loving-kindness (mettā), compassion (karuṇā), empathetic joy (muditā), and equanimity (upekkhā).

The Four Immeasurable Minds are cultivated in sequence, where loving-kindness is the foundation for developing compassion, which leads to empathetic joy, culminating in equanimity (14). These virtues enhance emotional and ethical well-being, reinforcing prosocial behavior such as kindness, caregiving, social harmony, and fairness in decision-making. Buddhist doctrinal sources suggest that contemplating death (Maranasati) fosters the cultivation of the Brahmavihara by instilling a sense of urgency and non-attachment. This contemplation can deepen compassion (*karuṇā*) and equanimity (*upekkhā*) as one reflects on the universality of death and the suffering of beings (15, 16). Additionally, understanding impermanence and the universality of suffering is said to strengthen loving-kindness and compassion (14, 17). While traditional teachings emphasize this relationship, empirical research directly examining the link remains limited. Although no studies have specifically explored the connection between death contemplation and the Four Immeasurable Minds, some research suggests that reflecting on mortality fosters the development of prosocial values. This aligns with perspectives from Meaning Management theory and other frameworks on mortality reflection, which propose that contemplating death reshapes one’s perception of life, leading to a deeper appreciation of meaningful relationships and personal growth (5, 12). Terror Management Theory similarly suggests that mortality reminders reinforce adherence to socially accepted cultural norms (18). Empirical findings offer partial support for these perspectives, showing that death meditation enhances multiple aspects of quality of life, including self-esteem and relationships, course (19), while death education programs increase empathy (7). Mortality salience (the awareness of one’s own inevitable death) manipulations tend to have also been linked to greater social acceptance of individuals with disabilities (20), increased generosity (21), and heightened helping behavior (22–24).

Research on Four Immeasurables meditation suggests that these practices can increase self-compassion and happiness (25) and foster positive self-perceptions and a sense of meaning in life (26). Studies on appreciative joy suggest positive associations with altruism and well-being, though findings regarding its impact on envy remain inconclusive (27). Equanimity, however, has been linked to higher well-being and reduced depression among Thai older adults in long-term care facilities (28). Given the importance of social relationships in older adult well-being, developing the Four Immeasurables may enhance interpersonal connections (29–34).

Despite growing interest in these associations, critical gaps remain in understanding how Buddhist death attitudes influence well-being, particularly through the cultivation of the Four Immeasurables. The current study aimed to explore whether (1) positive death attitudes predicted greater psychological well-being, and (2) if the development of the Four Immeasurables mediated this relationship. By examining Thai Buddhist older adults, this research contributes to spiritually integrated approaches to aging while providing empirical validation of Buddhist psychological models in a non-Western context.

## Materials and methods

### Study population and sample

The current study recruited participants among older adult Thai Buddhist meditation practitioners. Participant recruitment was conducted in Northern Thailand between August and October 2024. The final sample consisted of 332 participants recruited from temples and meditation centers across four provinces: Chiang Mai, Lampang, Mae Hong Son, and Uthai Thani. The following inclusion criteria were used for selecting the samples: age 60 and above; can read, write, and understand the Thai language; Theravada Buddhist; report having practiced death contemplation previously. Participants with uncorrected visual and/or hearing impairments were excluded from the study. Purposive sampling was used to ensure familiarity with the concepts of death and related practices from the perspective of Theravada Buddhism.

Sample size was calculated for linear regression with a small effect size, power (beta) set to 0.8 and significance level (alpha) of 0.05. The sample size required for the analysis was 311, and the total sample of 332 participants was included in the analysis.

### Procedure

A Thai-speaking research assistant contacted temples and meditation centers across Northern Thailand. Phone numbers were obtained online from publicly available resources, such as a meditation center’s website or social media page. The research assistant provided information about the study’s focus (death-related teachings and practices) and inquired about the data collection process. If the location administrators approved data collection and the site provided a suitable space, appropriate forms were provided (including a permission letter stating ethical approval of the research, participant information sheets (PIS), informed consent forms, compensation receipts, and a sample of the questionnaire) to ensure commitment to ethical practices. Data collection was then conducted on an agreed-upon date and led by Thai-speaking research assistants. On-site data collection took place in designated areas in temples or meditation centers. Information about the study was also distributed online with the possibility of online participation (Microsoft Forms survey). However, a majority of the sample was recruited on-site with paper questionnaires. Informed consent was obtained from all participants in the study.

The measures employed in this study were selected for their cultural relevance and suitability for the target population. The Morana Questionnaire, used to assess positive death attitudes, captures key aspects of maranasati as outlined in Theravada Buddhism, such as frequent contemplation of death, preparedness, and acceptance of death as a natural part of life. The Four Immeasurables Scale was chosen for its alignment with the constructs of loving-kindness, compassion, appreciative joy, and equanimity, which correspond directly to the Brahmaviharas in the Theravada tradition. To evaluate well-being, the WHO-5-T, a brief and internationally validated instrument, was employed. Additionally, the ZKA-20, a concise measure of the alternative five-factor model of personality, was utilized to reduce participant burden while maintaining psychometric robustness.

The data collection procedure was conducted as follows. First, participants were informed about the study’s focus and purpose, its voluntary nature, and their right to withdraw at any time without repercussions. This information was provided in the participant information sheets, and informed consent was obtained; a Thai-speaking research assistant then summarized the agreement. Second, informed consent was obtained from all participants (either in written form or online; the survey was only accessible to those who consented to participation). Third, the questionnaires were distributed (or displayed online). Upon completion of the questionnaire, a research assistant would check the completion of the questionnaire and provide options for compensation (receive in cash [on-site only], receive via bank transfer/QR payment, or do not receive compensation). Participants were compensated with 100 Thai baht each (approximately $3 US as of May 31, 2025). The primary investigator supervised data collection either on-site or virtually, when an on-site visit was not feasible.

### Ethical considerations

This study was approved by the Research Ethics Committee, Faculty of Medicine, Chiang Mai University. Research ID: PSY-2567-0372 on August 9th, 2024. As mentioned in the previous section, all participants were properly informed about the nature of the study and their rights (such as the right to withdraw), and informed consent was obtained from all participants. Paper questionnaires will be stored securely for 5 years in a locked cabinet at the Faculty of Medicine, Chiang Mai University. Digitalized data can be accessed only by research team members and are password-protected. All possible identification markers that may be used to trace questionnaires to participants have been removed. For example, informed consent forms and compensation receipts are kept separately from the questionnaires. Bank account information for those choosing to receive compensation via bank transfer was deleted upon confirmation of receipt of the transfer.

### Instruments

Sociodemographic information about age, gender, employment, and education was collected. The following instruments were used to measure positive death attitudes, well-being, and prosocial values.

#### Morana questionnaire

This measure consists of 10 items on a 5-point Likert scale (35). The measure assesses the frequency of thinking about death, calmness associated with thoughts about death, preparedness for death to come, understanding death as a natural part of life, and beliefs in the afterlife. The first nine items, for example, “I am ready to leave this world now, even though there is still much to do” (item 5), are scored from ‘strongly disagree’ (1) to ‘strongly agree’ (4). Item 10 began with a prompt “If I were to leave this world, I would want to go to.” with possible response options including: (1) Heaven/Higher Heaven, (2) Stay with those who respect and believe in you, (3) Keep Watching over those whom you care about, (4) be reborn as a new person. Since item 10 is categorical, it was not included in the summing up of the scores. Full scale can be found online. The items demonstrated good internal consistency in the current sample, with a Cronbach’s alpha of 0.894.

#### WHO well-being index (WHO-5)

The Thai version of the WHO Well-being Index (WHO-5-T) was used to assess subjective well-being (36). It consists of five items rated from 0 (never) to 5 (always), with the lower score representing lower well-being. Example item: “I feel calm and relaxed.” (item 2). The possible total scores range from 0 to 25; however, it is common among researchers to multiply the total score by 4 and provide a total out of 100. The scale demonstrated good internal consistency among the current sample, with a Cronbach’s alpha of 0.925.

#### Fi-12

The Four Immeasurable Scale (FI-12) is a 12-item questionnaire with a 7-point Likert scale, developed to measure loving-kindness, compassion, sympathetic joy, and equanimity (37). The response options range from ‘not at all like me’ (1) to ‘very much like me’ (5). Example items are: “It comes naturally for me to show kindness to others.” (item 5), “I congratulate those who have accomplished something great in their lives” (item 11). The full scale can be accessed online. In the current sample, the scale demonstrated good internal consistency, with Cronbach’s alpha equal to 0.952.

#### ZKA-20

A short version of the Zuckerman-Kuhlman-Aluja Personality Questionnaire (38) was used to assess personality traits, including extraversion, neuroticism, aggression, activity, and sensation-seeking. A shortened version was used to minimize the burden on the participants. This scale comprises 20 questions, each on a four-point Likert scale. Response options range from ‘strongly disagree’ (1) to ‘strongly agree’ (4). Each subscale showed adequate internal consistency, with Cronbach’s alphas as follows: aggression = 0.829, sensation-seeking = 0.817, activity = 0.750, extraversion = 0.831, and neuroticism = 0.854.

### Statistical analysis

Descriptive analyses were used for all variables in the study. Correlations were drawn between all variables. Hierarchical regression analysis was employed to investigate the hypothesis that positive death attitudes and the Four Immeasurable Minds are positively correlated with well-being. Mediation analysis was used to further test the potential mediating effects of the Four Immeasurable Minds on the relationship between positive death attitudes and well-being. A *p*-value below 0.05 was considered significant. IBM Statistical Package for Social Sciences (SPSS 26) and PROCESS were used for the analysis.

## Results

Table 1 presents the sociodemographic characteristics of the sample (*n* = 332). Most participants were female (66%) and unemployed (69.6%). The mean age of the participants was 68.12 (± 6.86).

**Table 1:** Demographic information and characteristics (*n* = 332).

Variables	Number	Percent
Gender		
Female	219	66
Male	113	34
Education		
> High school	165	49.7
≤ High school	167	50.3
Employment		
Employed	101	30.4
Unemployed	231	69.6

Table 2 presents the mean scores of the instruments used. The mean score of positive death attitudes was moderately high. Participants had a high level of well-being with a mean score of 75.96, well above the commonly accepted threshold of 50 for poor mental health. Participants also showed moderately high levels of the Four Immeasurable Minds.

**Table 2:** Positive death attitudes and mental health instrument scores (*n* = 332).

Instruments	Mean (SD)
MoQ (9–36)	29.66 (4.45)
WHO-5-T (0–25)/(0–100)	18.99 (4.65)/75.96
FI-12 (12–60)	47.05 (8.37)
ZKA-20	
ZKA extraversion (4–16)	12.63 (2.05)
ZKA neuroticism (4–16)	8.57 (2.92)
ZKA activity (4–16)	12.69 (2.14)
ZKA aggression (4–16)	7.86 (3.08)
ZKA sensation-seeking (4–16)	7.11 (2.05)

SD = standard deviation, MoQ = Morana Questionnaire, WHO-5-T = WHO Well-being Index Thai, FI-12 = Four Immeasurables Scale, ZKA-20 = Zuckerman-Kuhlman-Aluja Personality Questionnaire.

**Figure 1:**
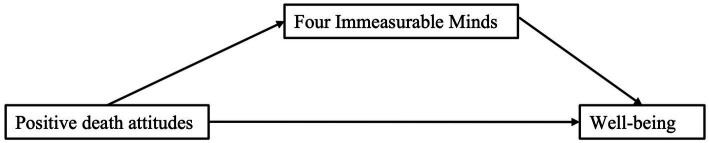
Conceptual mediation model.

Correlation analysis revealed significant positive associations between positive death attitudes, well-being, and Four Immeasurable Minds (Table 3; Figure 1).

**Table 3:** Correlation analysis revealed significant positive associations between positive death attitudes, well-being, and the Four Immeasurable Minds.

Items		1	2	3	4	5	6	7	8	9	10	11	12
1	Age	–											
2	Gender	−0.108*	–										
3	Education	−0.098	0.002	–									
4	Employ	0.181**	−0.119*	−0.212**	–								
5	Death attitudes	0.084	−0.041	0.095	−0.056	–							
6	Well-being	−0.057	0.02	0.104	−0.029	0.385**	–						
7	FI-12	−0.024	−0.024	0.142**	0.03	0.420**	0.420**	–					
8	Extraversion	−0.096	−0.005	0.181**	0.000	0.314**	0.330**	0.444**	–				
9	Neuroticism	0.016	−0.039	0.031	−0.011	−0.233**	−0.401**	−0.270**	−0.066	–			
10	Activity	−0.071	0.005	0.181**	0.012	0.332**	0.290**	0.493**	0.695**	−0.072	–		
11	Aggression	−0.040	0.065	−0.07	−0.002	−0.309**	−0.259**	−0.249**	−0.065	0.715**	−0.082	–	
12	Sensation-seeking	0.149**	−0.085	−0.188**	−0.088	−0.314**	−0.340**	−0.498**	−0.592**	0.180**	−0.676**	0.171**	–

*p < 0.05, **p < 0.01. FI-12 = Four Immeasurables Scale.

**Figure 2:**
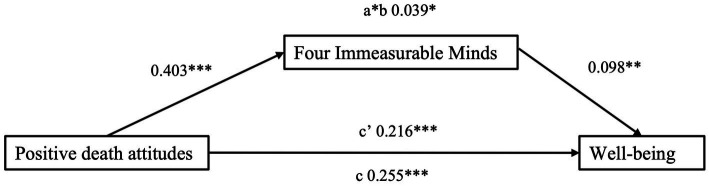
Unstandardized effects of the mediation analysis.

The non-mediated regression model of positive death attitudes predicting well-being, accounting for sociodemographic characteristics (age, gender, education, employment) and personality traits, was significant, *F*(10,321) = 15.316, *p* < 0.001 (Table 4). Positive death attitudes significantly and positively predicted well-being, B (unstandardized) = 0.255, β = 0.244, se = 0.055, *p* < 0.001. The model could explain 30.2% of the variance in well-being. Mediation analysis was used to test the mediating effects of Four Immeasurable Minds on the relationship between positive death attitudes and well-being. Positive death attitudes had a positive effect on Four Immeasurable Minds, B (unstandardized) = 0.403, β = 0.215, se = 0.094, *p* < 0.001, and well-being, B (unstandardized) = 0.216, β = 0.207, se = 0.055, *p* < 0.001 (Figure 2). Four Immeasurable Minds significantly affected well-being, B (unstandardized) = 0.098, β = 0.175, se = 0.032, *p* = 0.003. The indirect effect of positive death attitudes on well-being via Four Immeasurable Minds was significant, B (unstandardized) = 0.039, β = 0.038, LLCI = 0.1069, ULCI = 0.3250, 95% CI. The model could explain 34.2% of the variance in well-being. A summary of the total, direct, and indirect effects of positive death attitudes on well-being can be found in Table 5.

**Table 4:** Summary of regression of positive death attitudes predicting well-being, controlling for age, gender, education, employment, and personality (*n* = 332).

Antecedent	Coeff (B)	SE	β	*p*-value
X (Death attitudes)	0.255	0.055	0.244	**<0.001**
Age	−0.017	0.033	−0.025	0.602
Gender	−0.082	0.462	−0.008	0.859
Edu	0.383	0.459	0.041	0.404
Employ	−0.182	0.497	−0.018	0.714
Extra	0.334	0.154	0.147	**0.031**
Neuro	−0.682	0.106	−0.429	**<0.001**
Act	−0.020	0.155	−0.009	0.896
Aggr	0.224	0.103	0.148	**0.031**
Sens	−0.294	0.158	−0.130	0.064
Constant	14.952	3.947		**<0.001**
R^2^ = 0.323				
*F*(10,321) = 15.316, *p* < 0.001				

MoQ = Morana Questionnaire, Coeff (B) = unstandardized beta coefficient, SE = standard error, β = standardized beta coefficient, edu = education, employ = employment, extra = extraversion, neuro = neuroticism, act = activity, aggr = aggression, sens = sensation-seeking. *Significant values (*p* < 0.05) highlighted in bold.

**Table 5:** Total, direct, and indirect effects of positive death attitudes on well-being.

Pathway	β	B	SE	LL95%CI	UL95%CI
Death attitudes ➔ Well-being (c, total)	0.244	0.255	0.055	0.148	0.363
Death attitudes ➔ FI (a)	0.215	0.403	0.094	0.219	0.587
FI ➔ Well-being (b)	0.175	0.098	0.032	0.034	0.161
Death attitudes ➔ Well-being (ab, indirect)	0.038	0.039	0.018	0.007	0.079
Death attitudes ➔ Well-being (c’, direct)	0.207	0.216	0.055	0.107	0.325

FI = Four Immeasurable Minds, B = unstandardized beta coefficient, β = standardized beta coefficient, SE = standard error, LL = lower level, UL = upper level, CI = confidence interval.

## Discussion

To our knowledge, this is one of the first studies assessing the effects of positive death attitudes on well-being in a Buddhist context. The participants exhibited a moderately high level of positive attitudes toward death. It should be noted that all participants were practicing Buddhists, with Buddhist practices having previously been found to be associated with lower death anxiety and higher death acceptance (39).

The results confirmed the study’s hypotheses. Positive death attitudes are positively associated with well-being, and the Four Immeasurable Minds mediated the relationship. The findings confirm that positive death attitudes are a beneficial mindset contributing to positive mental health. Acceptance and reflection of death can promote motivation for personal growth and for building meaningful relationships (12), leading to an increase in prosocial values and behaviors. The findings support Meaning-Management Theory, which suggests that accepting one’s death can lead to meaning-making by cultivating one’s values and identity. Previous research on Terror Management Theory found that manipulations of mortality awareness can increase prosocial behavior, particularly towards the ingroup (40). In the current study, positive death attitudes, characterized by acceptance and frequent contemplation, were associated with higher prosocial virtues. While the current study provided a theoretical foundation and initial empirical support for the association between the two variables, further investigation is needed to establish a strong empirical basis, examining the relationship more directly. Prosocial behavior and positive social relationships may also create a sense of purpose and productivity, which is particularly important to older people who are likely to have lost work and/or social roles, contributing to positive self-evaluations, shifting attention away from the negative aspects of life, instilling meaning in life, and facilitating positive integration (41). Prosociality tends to be higher among older adults, likely due to an increased perception that life as finite and that they should prioritize emotional goals, such as personal connections (42). In line with this idea, a qualitative study found that older Thai individuals who experienced major losses during the 2004 tsunami engaged in community service to enhance their self-esteem, highlighting that doing good things for others was more meaningful than simply doing good things for themselves (43).

Positive death attitudes also directly affected well-being, consistent with previous research and the authors’ hypothesis. Accepting and reflecting on death can transform one’s perspective on life, shifting focus to the positive aspects of it (5, 12). Four Immeasurable Minds mediated the relationship between positive death attitudes and well-being, explaining a 13.24% increase in variance compared to the non-mediated regression model. This suggests that thinking and behaving in a prosocial manner, consistent with Buddhist teachings, are essential components of well-being in the current sample. Both positive death attitudes and prosocial values (the Four Immeasurable Minds) contributed to the sense of well-being among older Thai Buddhists.

## Research implications

This study has several academic and societal implications. It contributes to the limited research on positive attitudes towards death and the four immeasurable, providing support for these as positive psychological factors that may enhance well-being. These findings can be used to develop care plans and recommendations tailored to the needs of older adults. Positive death attitudes were associated with higher levels of the four immeasurable minds, which may protect against loneliness, a common problem in the context of social role losses faced by older adults. Given the global trend of an ageing population, this study offers valuable insights into the importance of positive death attitudes for the well-being of older people. Though the cross-sectional nature of this study does not establish causality, it may serve as an initial foundation for integrating positive attitudes about death into mental health promotion programs. Components of positive death attitudes, such as accepting death as natural and feeling prepared for it both psychologically and materially can be isolated to develop programs for non-Buddhists. This may be similar to the way Buddhist practices (e.g., mindfulness meditation) have been adapted extensively in the West without specific requirements regarding Buddhist affiliations and familiarity with other practices (44). As such, Buddhist teachings about death can provide a guide and framework for programs aimed at cultivating positive attitudes about death, possibly in the form of an education course.

Though the Four Immeasurable Minds are rooted in Buddhist teachings, previous research has demonstrated that these meditations may provide beneficial effects when used outside of the religious context (45–47). It is essential to note that such adaptations necessitate careful consideration and consultation with relevant experts to ensure adherence to ethical principles and to avoid oversimplification or overreduction. Adapting death meditation practice outside of a Buddhist context may pose difficulties if people view death in a drastically different way (e.g., perceiving death as a taboo topic), have recently experienced a loss, or have diagnosed psychiatric conditions such as major depressive disorder. In these cases, introducing death meditation without a Buddhist foundation may lead to harmful consequences, with an exacerbation of depressive symptoms.

Our findings may also help in understanding how the growing volume of death-related media can affect public health, although this issue is beyond the scope of the current research.

## Strengths

The current study has provided an initial foundation for investigation of the effects of positive beliefs and attitudes about death, their impact on well-being, and the possible mediating effects of the Four Immeasurable Minds. Testable hypotheses can be generated based on the findings of the current study. Additionally, this study can be replicated both directly and conceptually. Given the cross-sectional nature of this study, replications can be conducted inexpensively and quickly. This study imposed a minimal burden on the participants and posed minimal risks, making ethical violations unlikely.

A significant strength of this study lies in its pioneering examination of how positive beliefs about death, shaped by Buddhist death contemplation practices, impact well-being among older Thai Buddhists, both directly and through the cultivation of the Four Immeasurable Minds. By utilizing culturally relevant and validated measures with a large sample, this research offers essential and original insights into the psychological resources that support healthy aging in a non-Western context. The rigorous mediation analysis and careful control of confounding variables further strengthen the validity of the findings.

Importantly, as the first study of its kind, these findings highlight the need for further research. Future studies should investigate the effectiveness of mindfulness-based interventions that promote positive attitudes towards death and the Four Immeasurable Minds, both within Buddhist communities and across diverse cultural contexts. Longitudinal and experimental research are also recommended to establish causality and assess long-term impacts on psychological resilience and well-being in older adults.

## Limitations

The current study has several limitations. First, its cross-sectional nature limits its ability to establish causal relationships. A longitudinal study could address this limitation. Second, the study recruited only Thai Buddhist meditation practitioners, limiting its generalizability to other populations. Further research could attempt to replicate the results with younger participants, those without meditation experience, and possibly practitioners from other religions or cultures.

## Conclusion

Positive death attitudes positively affected well-being among Thai older meditation practitioners, and Four Immeasurable Minds mediated the relationship. These findings demonstrate the beneficial effects of positive attitudes about death, consistent with Buddhist teachings, and the positive effects of prosocial virtues and behaviors reflected in the Four Immeasurable Minds. The results may be used to devise mental healthcare programs and tackle public health issues related to geriatric well-being.

## Data Availability

The raw data supporting the conclusions of this article will be made available by the authors, without undue reservation.
